# Prognostic Nutritional Index as a Predictor of Surgical Morbidity in Total Neoadjuvant Therapy for Locally Advanced Rectal Cancer

**DOI:** 10.3390/jcm14061937

**Published:** 2025-03-13

**Authors:** Cem Batuhan Ofluoğlu, Fırat Mülküt, İsa Caner Aydın, Mustafa Kağan Başdoğan, İbrahim Aydın

**Affiliations:** 1 Department of Gastrointestinal Surgery, Sancaktepe Şehit Prof. Dr. İlhan Varank Training and Research Hospital, University of Health Sciences, Istanbul 34785, Turkey; 2 Department of General Surgery, Sancaktepe Şehit Prof. Dr. İlhan Varank Training and Research Hospital, University of Health Sciences, Istanbul 34785, Turkey; firatmulkut@hotmail.com (F.M.); mustafabasdogan@gmail.com (M.K.B.); ibrahimaydin061@gmail.com (İ.A.); 3 Department of Gastrointestinal Surgery, Ministry of Health Zonguldak Ataturk State Hospital, Zonguldak 67100, Turkey; isacaneraydin@outlook.com

**Keywords:** total neoadjuvant therapy, rectal cancer, surgical complications, prognostic nutritional index, anastomotic leakage, perioperative morbidity

## Abstract

**Background:** The management of locally advanced rectal cancer (LARC) has seen the emergence of total neoadjuvant therapy (TNT) as a promising approach. TNT has shown potential in enhancing tumor regression, increasing pathological complete response (pCR) rates, and improving the control of systemic disease. However, the impact of TNT on complications during and after surgery remains uncertain. This research aimed to assess surgical complications linked to TNT in comparison with conventional neoadjuvant chemoradiotherapy (nCRT). Additionally, this study explored the potential of the Prognostic Nutritional Index (PNI) as a predictor of surgical outcomes. **Methods:** A retrospective cohort study was conducted at Sancaktepe Şehit Prof. Dr. İlhan Varank Training and Research Hospital, including patients with LARC who underwent either TNT or nCRT followed by curative excision (TME). Demographic data, perioperative complications, and tumor-related variables were also analyzed. The prognostic value of the PNI in predicting surgical complications was assessed using multivariate logistic regression analysis. Statistical significance was set at *p* < 0.05. **Results:** A total of 103 patients with LARC were included, of whom 38 (36.9%) received TNT and 65 (63.1%) underwent nCRT. TNT was associated with significantly higher rates of anastomotic leakage (13.2% vs. 6.2%, *p* = 0.04) and wound infections (23.7% vs. 9.2%, *p* = 0.02). The mean tumor size was significantly smaller in the TNT group (3.22 ± 1.10 cm) than in the nCRT group (3.65 ± 1.26 cm, *p* = 0.02). The PNI was significantly lower in the TNT group (38.96 ± 5.54) than in the nCRT group (41.31 ± 4.65, *p* = 0.03). Multivariate logistic regression analysis demonstrated that a lower PNI was independently associated with increased surgical complications (β = −1.09, *p* = 0.028, 95% CI: −2.06–−0.12). **Conclusions:** Although TNT demonstrates clear oncological benefits in LARC, it is associated with increased perioperative morbidity. Our findings suggest that the PNI is a valuable predictive biomarker of surgical complications in patients treated with TNT. Preoperative nutritional assessment and optimization may improve perioperative outcomes and mitigate the risks associated with TNT. Future prospective studies should explore targeted interventions to enhance the safety profile of TNT while preserving its oncological advantages.

## 1. Introduction

A significant proportion of colorectal malignancies, specifically 30% of all diagnosed cases and approximately 70% of rectal cancer cases, fall under the classification of locally advanced rectal cancer (LARC) [1]. This particular stage of rectal cancer poses considerable clinical complexities, often necessitating a multidisciplinary treatment approach to optimize disease management. LARC is typically defined by tumors staged as T3–T4 or cases presenting with positive lymph node involvement, indicating a more aggressive disease course that requires preoperative interventions aimed at improving surgical outcomes and long-term prognosis.

Traditionally, the standard treatment paradigm for LARC consisted of neoadjuvant chemoradiotherapy (nCRT) followed by total mesorectal excision (TME) [2]. The rationale behind this two-step therapeutic strategy is to enhance local tumor control, minimize recurrence risk, and improve the feasibility of complete surgical resection. While nCRT has demonstrated significant oncological benefits, including tumor downstaging and improved resectability, it is not without its limitations. The approach has been associated with suboptimal tumor regression, increased recurrence rates, and a delay in the initiation of systemic therapy, which may negatively impact long-term disease control [1,3,4]. To address these shortcomings, total neoadjuvant therapy (TNT) has emerged as an alternative approach that integrates systemic chemotherapy before and after radiotherapy [5,6,7]. The primary goal of TNT is to enhance systemic disease control, facilitate more effective tumor shrinkage, and improve the likelihood of achieving a pathological complete response (pCR). This treatment modification has been rigorously evaluated in randomized controlled trials, which have collectively demonstrated that TNT leads to superior disease-free survival (DFS) rates and higher treatment adherence among patients undergoing therapy [8,9]. Despite its promising oncological benefits, TNT remains a relatively novel therapeutic strategy, and the available clinical data regarding its long-term effectiveness and perioperative safety profile remain somewhat limited. A critical aspect of optimizing TNT outcomes is the identification of reliable prognostic biomarkers that can predict surgical morbidity and treatment-related toxicities [10,11]. One such biomarker that has gained attention in recent years is the Prognostic Nutritional Index (PNI). This index is an important parameter in oncology as it provides an assessment of a patient’s immunonutritional status, which is determined based on serum albumin levels and total lymphocyte count.

The PNI has been extensively studied in various oncological settings and has been recognized for its role in predicting postoperative complications, overall survival (OS), and DFS. Numerous studies have indicated that lower PNI levels are correlated with poorer oncological outcomes, increased perioperative morbidity, and extended hospital stays following surgical intervention. In the context of gastrointestinal malignancies, including rectal cancer, a diminished PNI has been strongly linked to anastomotic leakage, impaired wound healing, and a heightened risk of postoperative infections. Furthermore, in the neoadjuvant setting, patients with low PNI values have exhibited reduced chemotherapy tolerance and heightened susceptibility to treatment-related toxicities, thereby compromising overall treatment efficacy [11,12]. Given that TNT is known to exert immunosuppressive effects, a patient’s nutritional and immune status may play a pivotal role in determining post-treatment recovery and surgical outcomes. As such, there is a growing emphasis on the importance of preoperative nutritional assessment and intervention strategies aimed at optimizing patient health before initiating TNT. Future research should focus on developing personalized treatment protocols that incorporate PNI-driven risk stratification, enabling clinicians to tailor interventions that enhance treatment efficacy while minimizing potential complications [11,12].

This study aimed to evaluate the perioperative outcomes of TNT in patients with LARC with a primary focus on its impact on surgical morbidity, postoperative complications, and short-term recovery metrics. Additionally, this study explored potential prognostic factors, including the PNI, to assess their role in predicting surgical outcomes and treatment-related adverse effects.

Understanding the role of the PNI may help optimize patient selection and perioperative management strategies in TNT-treated LARC cases.

## 2. Materials and Methods

### 2.1. Study Design and Patient Selection

This retrospective cohort study was conducted at Sancaktepe Şehit Prof. Dr. İlhan Varank Training and Research Hospital and included patients diagnosed with LARC between January 2018 and January 2024. LARC was defined as clinical stage T3–4 and/or node-positive disease. The study population consisted of patients who underwent either TNT or standard nCRT, followed by curative-intent surgery. Patients were divided into two groups based on their treatment protocol: those receiving TNT and those managed with conventional nCRT. Data were collected through a comprehensive review of electronic medical records, including demographic information, tumor characteristics, treatment details, and perioperative outcomes.

Patients eligible for inclusion were those who had histopathologically confirmed LARC, completed their assigned neoadjuvant treatment regimen, or underwent a TME. The exclusion criteria included patients younger than 18 years or older than 80 years, those with metastatic (M1) disease at diagnosis, individuals requiring emergency surgical intervention, and patients with incomplete staging or missing clinical data. Patients who achieved clinical complete response (cCR) and were managed with a watch-and-wait (WAW) strategy were excluded because they did not undergo surgical intervention.

### 2.2. Treatment Protocols

The TNT protocol used in this study was designed in accordance with the NCCN 2024 Guidelines for LARC. The treatment regimen consists of long-course chemoradiotherapy (CRT) followed by systemic chemotherapy before a curative-intent TME (16). Neoadjuvant chemoradiotherapy (CRT): patients received external beam radiotherapy (RT) over 5–6 weeks (25–30 fractions) at a total dose of 50.4 Gy, delivered concurrently with capecitabine (825 mg/m^2^, twice daily, on days 1–5 of each week). Consolidation chemotherapy: after the completion of CRT, patients received 4–6 cycles of systemic chemotherapy using the CAPOX regimen, consisting of capecitabine (1000 mg/m^2^, days 1–14) plus oxaliplatin (130 mg/m^2^, day 1), administered every 21 days. After the completion of neoadjuvant therapy, surgery was performed within 6–8 weeks, following standard oncological and surgical principles.

### 2.3. Surgical and Postoperative Management

All patients underwent TME performed by experienced colorectal surgeons. The choice of surgical approach, either laparoscopic or open, was determined based on tumor location, clinical response, and surgeon preference. Intraoperative data, including operative time and the need for conversion to open surgery, were recorded. Anastomotic integrity was assessed intraoperatively using the rectal air leak test. Ileostomy was selectively performed based on the surgical judgment. Postoperative care followed standardized institutional protocols, including thromboembolic prophylaxis, antibiotic administration, and early mobilization. Patients were followed up at regular intervals to assess short-term surgical outcomes and postoperative recovery.

### 2.4. Data Collection and Outcome Measures

The primary endpoints of this study were perioperative outcomes, including postoperative morbidity and complication rates. Major complications, including anastomotic leakage, ileus, and wound infections, were analyzed in both groups. The 30-day morbidity rate was also reported.

Secondary endpoints included the incidence of anastomotic leakage, ileus, and wound infections.

The prognostic role of the PNI in predicting surgical outcomes and postoperative complications was also analyzed. The PNI was calculated using the standard formula: PNI = (10 × serum albumin level [g/dL]) + (0.005 × total lymphocyte count [per mm^3^]). The PNI was assessed preoperatively as part of the routine perioperative evaluation.

### 2.5. Ethical Considerations

This research was carried out in line with the ethical guidelines set in the Helsinki Declaration and received approval from the Scientific Research Ethics Committee of Sancaktepe Şehit Prof. Dr. İlhan Varank Training and Research Hospital. The ethical clearance was granted under protocol number 26, with the approval date being 22 January 2025.

### 2.6. Statistical Analysis

Patient demographics, tumor characteristics, and clinical outcomes were summarized using descriptive statistics. For continuous variables, means with standard deviations or medians with interquartile ranges were reported. Frequencies and percentages were used to present categorical variables. Comparisons between the TNT and nCRT groups were performed using Student’s *t*-test or Mann–Whitney U test for continuous variables and the Chi-square test or Fisher’s exact test for categorical variables. Multivariate logistic regression analysis was conducted to assess the independent impact of the PNI on surgical morbidity and postoperative complications. Statistical significance was set at a two-tailed *p*-value of <0.05. All statistical analyses were performed using SPSS version 25.0 (IBM Corp., Armonk, NY, USA).

## 3. Results

The research involved 103 patients diagnosed with LARC. Of these, 38 (36.9%) underwent TNT treatment, while 65 (63.1%) received CRT. The overall cohort had a mean age of 62.6 ± 11.6 years, with ages spanning from 35 to 81 years. Gender distribution showed 62 (60.2%) males and 41 (39.8%) females.

No significant differences were observed between the TNT and CRT groups in terms of mean age, gender distribution, BMI scores, ASA scores, and chronic disease prevalences (*p* = 0.26, *p* = 0.57, *p* = 0.64, *p* = 0.81, and *p* > 0.05 respectively).

The TNT group experienced significantly longer operative times (251.0 ± 54.0 min) compared to the CRT group (218.2 ± 34.2 min, *p* = 0.04). Laparoscopic surgery was slightly less common in the TNT group (57.9%) than in the CRT group (61.5%), though this difference was not statistically significant (*p* = 0.07).

Regarding perioperative complications, the TNT group showed a higher rate of conversion to open surgery (21.1%) compared to the CRT group (6.2%, *p* = 0.05), with borderline statistical significance. Anastomotic leakage occurred significantly more often in the TNT group (13.2%) than in the CRT group (6.2%; *p* = 0.04). The TNT group also experienced a significantly higher rate of wound infections (23.7%) compared to the CRT group (9.2%, *p* = 0.02).

Ryan TRS and pCR did not differ significantly between the groups (*p* = 0.35, *p* = 0.68, respectively). The mean tumor size was significantly smaller in the TNT group (3.22 ± 1.10 cm) than in the CRT group (3.65 ± 1.26 cm, *p* = 0.02) (Table 1).

A correlation analysis was conducted to evaluate the relationship between the PNI and surgical outcomes, including operative time, wound infection, postoperative ileus, anastomotic leakage, and T stage downstaging. A significant negative correlation was observed between the PNI and operative time (r = −0.215, *p* = 0.047). Wound infection showed a borderline significant positive correlation with the PNI (r = 0.199, *p* = 0.067). Other surgical outcomes, including postoperative ileus (r = −0.041, *p* = 0.710), anastomotic leakage (r = 0.110, *p* = 0.312), and T stage downstaging (r = −0.077, *p* = 0.481), were not significantly correlated with the PNI (Table 2).

The multivariate logistic regression analysis showed that TNT was associated with an increased risk of surgical complications (β = 0.69, *p* = 0.19); however, the difference was not statistically significant. The PNI (as a continuous variable) was significantly associated with a lower risk of surgical complications (β = −0.10, *p* = 0.05). Analysis of the PNI as a categorical variable (PNI ≥ 40 vs. PNI < 40) showed that PNI ≥ 40 was significantly associated with a lower risk of surgical complications (β = −1.09, *p* = 0.028, 95% CI: −2.06–−0.12). BMI was associated with increased surgical complications (β = 0.08, *p* = 0.15). DM was not significantly associated with surgical complications (β = 0.28, *p* = 0.63) (Table 3).

## 4. Discussion

Total neoadjuvant therapy has become an increasingly used strategy in the management of LARC, offering superior tumor downstaging, increased pCR rates, and enhanced systemic disease control [1,3,7]. Our findings are consistent with these oncological advantages, as the TNT group in our study demonstrated significantly smaller tumor sizes at the time of surgery than the nCRT group. However, this benefit comes at the cost of increased perioperative complications, particularly higher rates of anastomotic leakage, wound infections, and conversion to open surgery. These findings align with previous reports suggesting that, while TNT optimizes tumor regression, it may also predispose patients to greater surgical morbidity [4,5,6].

Several mechanisms may explain the increased risk of complications after TNT. Extended chemotherapy exposure in TNT patients can lead to immunosuppression, impaired tissue repair, and endothelial dysfunction, which in turn may contribute to poor wound healing and an increased likelihood of infectious complications [3,13]. Additionally, radiotherapy-induced fibrosis within the mesorectum may increase technical difficulties during TME, leading to a higher risk of anastomotic leakage [4,14]. The RAPIDO and PRODIGE-23 trials, which assessed the efficacy of TNT in LARC, similarly reported increased surgical morbidity in TNT-treated patients, emphasizing the need for perioperative risk-mitigation strategies [15].

A novel aspect of our study is the investigation of the PNI as a predictor of surgical outcomes in TNT-treated patients. Our results indicate that patients in the TNT group had significantly lower preoperative PNI scores than those in the nCRT group. More importantly, lower PNI values were independently associated with a higher risk of postoperative complications, including anastomotic leakage and wound infections. This finding suggests that nutritional and immunological status may play a critical role in the surgical outcomes after TNT. Our study is among the first to explore this relationship in the context of TNT, distinguishing it from prior research that primarily focused on oncologic outcomes. Our results are consistent with recent findings that a low PNI is associated with increased perioperative complications in patients with LARC undergoing neoadjuvant therapy [12,16]. Moreover, studies emphasized the role of inflammatory and nutritional markers in predicting surgical outcomes in rectal cancer surgery [10,11,17,18]. Given these findings, our study underscores the importance of preoperative nutritional optimization as a potential intervention to mitigate surgical morbidity in TNT-treated patients.

Our findings emphasize the need for a personalized approach to TNT implementation, particularly in high-risk patients with a compromised immunonutritional status. Prolonged chemotherapy exposure in TNT leads to cumulative toxicity, resulting in immunosuppression, impaired wound healing, and an increased risk of surgical complications. This is reflected in the significantly lower PNI scores in TNT-treated patients. Given this risk, a preoperative PNI assessment could facilitate the early identification of high-risk patients, allowing for targeted interventions such as nutritional support, immunonutritional therapies, and individualized perioperative care. Recent studies have demonstrated that lower PNI levels predict increased postoperative morbidity in rectal cancer patients undergoing neoadjuvant therapy, reinforcing the need for structured perioperative nutritional and immunological optimization [9,19]. Considering the paradox of TNT-induced tumor regression alongside its immunosuppressive effects, future research should investigate the integration of immunomodulatory strategies, such as perioperative immune-enhancing nutritional support or checkpoint inhibitors in selected patients [20,21]. Incorporating nutritional screening and optimization protocols into TNT treatment algorithms may help balance oncological benefits with surgical risks. Further prospective studies should evaluate the effectiveness of structured nutritional interventions in improving surgical outcomes and mitigating complications in patients treated with TNT.

### Strengths and Limitations

Our study has several strengths. This study provides real-world data comparing TNT and nCRT in terms of surgical morbidity, offering valuable insights into perioperative risks in clinical settings. This study is one of the first to evaluate PNI as a prognostic biomarker in TNT-treated patients, adding a novel perspective to the growing body of literature on preoperative risk stratification.

However, this study has several limitations. The retrospective nature of this study introduces an inherent selection bias, which may have influenced the observed associations. Additionally, long-term oncological outcomes were not assessed, limiting the ability to evaluate the impact of TNT on long-term prognosis. One limitation of our study is the lack of stratified subgroup analyses based on key clinical factors, such as frailty status, comorbidities, and molecular tumor profiles. Future studies should incorporate these variables to determine which patient populations derive the greatest oncologic benefit from TNT while minimizing perioperative morbidity. As an exploratory study, our findings serve as a basis for further research, encouraging personalized treatment strategies tailored to individual patient profiles.

## 5. Conclusions

This study reinforces the oncological benefits of TNT in LARC and highlights its association with increased perioperative morbidity. Our findings demonstrate that the PNI is a significant predictor of surgical complications in TNT-treated patients, suggesting that preoperative nutritional assessment and optimization may play a crucial role in mitigating these risks. Patients with lower PNI scores exhibited a higher incidence of anastomotic leakage, wound infections, and prolonged hospital stay, emphasizing the need for targeted perioperative interventions.

Given the growing adoption of TNT in clinical practice, structured nutritional optimization strategies and immunomodulatory approaches should be explored to enhance patient outcomes while preserving the oncologic advantages of TNT. Future prospective studies are warranted to assess the impact of perioperative nutritional support, immune-enhancing interventions, and patient stratification models in order to improve surgical safety in TNT-treated patients. By integrating PNI-driven risk assessment into clinical decision-making, we can refine perioperative management strategies and optimize patient selection for TNT.

## Figures and Tables

**Table 1:** Comparison of patient demographics, perioperative outcomes, and tumor-related features in standard CRT and TNT groups.

Variables	Standard CRT Group (65)	TNT Group (38)	*p*-Value
Age (years)	61.6 (35.0–80.0), ±11.8	64.3 (38.0–81.0), ±11.3	0.26
Gender (male) (n, %)	41 (63.1%)	21 (55.3%)	0.57
BMI (mean, SD)	27.5 (18.6–37.7), ±5.0	27.1 (18.1–36.5), ±3.9	0.64
ASA (n,%)	34 (52.3%)	65 (171.1%)	0.81
DM (n, %)	38 (58.5%)	65 (171.1%)	1.00
COPD (n, %)	38 (58.5%)	65 (171.1%)	0.41
CAD (n, %)	38 (58.5%)	65 (171.1%)	0.25
HT (n, %)	38 (58.5%)	65 (171.1%)	0.88
PNI	41.31 (26.28–50.43), ±4.65	38.96 (24.96–45.69), ±5.54	0.03
Perioperative features			
Operative time (min) (mean, SD)	218.2 (150.0–315.0), ±34.2	251.0 (170.0–420.0), ±54.0	0.04
Surgical approach (laparoscopic) (n, %)	40 (61.5%)	22 (57.9%)	0.07
Perioperative complications			
Conversion to open surgery (n, %)	4 (6.2%)	8 (21.1%)	0.05
Anastomotic leakage (n, %)	4 (6.2%)	5 (13.2%)	0.04
Postoperative Ileus (n, %)	7 (10.8%)	6 (15.8%)	0.06
Wound infection (n, %)	6 (9.2%)	9 (23.7%)	0.02
Length of hospital stay (days, mean ± SD)	6.2 ± 2.1	7.8 ± 2.5	0.04
Tumor-related features			
Ryan TRS (mean ± SD)	2.28 ± 1.04	1.91 ± 1.14	0.35
pCR (n, %)	7 (10.8%)	6 (17.6%)	0.68
Tumor size (cm) (mean ± SD)	3.65 (1–6), ±1.26	3.22 (1–5.5), ±1.10	0.02
T stage (mean ± SD)	2.84 ± 0.72	2.75 ± 0.84	0.44
Extracted lymph nodes (mean ± SD)	12.78 ± 4.07	12.43 ± 3.36	0.29
Positive lymph nodes (mean ± SD)	1.3 ± 2.34	1.14 ± 2.11	0.91

*t*-tests, Chi-square tests. CRT: standard chemoradiotherapy, TNT: total neoadjuvant therapy, BMI: body mass index, ASA: American Society of Anesthesiologists (Physical Status Classification System), DM: diabetes mellitus, COPD: chronic obstructive pulmonary disease, CAD: coronary artery disease, HT: hypertension PNI: Prognostic Nutritional Index, TRS: tumor regression score (Ryan Scoring System), pCR: pathological complete response, SD: standard deviation, cm: centimeters.

**Table 2:** Correlation analysis between the PNI and surgical outcomes.

	PNI	Operative Time	Wound Infection	Postoperative Ileus	Anastomotic Leakage	T Stage Downstaging
PNI	1.00, -	−0.215, 0.047	0.199, 0.067	−0.041, 0.710	0.110, 0.312	−0.077, 0.481
Operative Time	−0.215, 0.047	1.00, -	0.08, 0.182	0.09, 0.154	0.07, 0.225	−0.12, 0.097
Wound Infection	0.199, 0.067	0.08, 0.182	1.00, -	0.06, 0.276	0.14, 0.113	0.04, 0.372
Postoperative Ileus	−0.041, 0.710	0.09, 0.154	0.06, 0.276	1.00, -	0.10, 0.205	−0.05, 0.318
Anastomotic Leakage	0.110, 0.312	0.07, 0.225	0.14, 0.113	0.10, 0.205	1.00, -	−0.03, 0.437
T stage Downstaging	−0.077, 0.481	−0.12, 0.097	0.04, 0.372	−0.05, 0.318	−0.03, 0.437	1.00, -

Pearson correlation; Spearman correlation; PNI: Prognostic Nutritional Index.

**Table 3:** Regression analysis of predictors for surgical complications in patients undergoing TNT or CRT.

Variable	Coefficient (β)	SE	z	*p*	95% CI
Intercept (constant)	−1.45	2.85	−0.51	0.61	(−7.03, 4.12)
TNT	0.69	0.52	1.32	0.19	(−0.33, 1.71)
PNI (continuous)	−0.10	0.05	−2.00	0.05	(−0.20, −0.00)
PNI ≥ 40	−1.09	0.49	−2.20	0.028	(−2.06, −0.12)
BMI	0.08	0.05	1.44	0.15	(−0.03, 0.18)
Diabetes	0.28	0.57	0.48	0.63	(−0.84, 1.39)

Multivariate logistic regression, TNT: total neoadjuvant therapy, CRT: standard chemoradiotherapy, PNI: Prognostic Nutritional Index, BMI: body mass index, SE: standard error, CI: confidence interval.

## Data Availability

The datasets generated and analyzed during the current study are available from the corresponding author upon reasonable request.
